# Recurrent cerebrovascular events after recent small subcortical infarction

**DOI:** 10.1007/s00415-024-12460-8

**Published:** 2024-05-27

**Authors:** Melanie Haidegger, Nina Klock, Markus Kneihsl, Simon Fandler-Höfler, Sebastian Eppinger, Kathrin Eller, Stephan Seiler, Christian Enzinger, Thomas Gattringer

**Affiliations:** 1 Department of Neurology, Medical University of Graz, Auenbruggerplatz 22, 8036 Graz, Austria; 2 Division of Neuroradiology, Vascular and Interventional Radiology, Department of Radiology, Medical University of Graz, Graz, Austria; 3 Division of Nephrology, Department of Internal Medicine, Medical University of Graz, Graz, Austria

**Keywords:** Cerebral small vessel disease, Recurrent events, Intracranial haemorrhage, Imaging, Recent small subcortical infarct

## Abstract

**Background:**

Recent small subcortical infarcts (RSSI) are the neuroimaging hallmark feature of small vessel disease (SVD)-related acute lacunar stroke. Long-term data on recurrent cerebrovascular events including their aetiology after RSSI are scarce.

**Patients and methods:**

This retrospective study included all consecutive ischaemic stroke patients with an MRI-confirmed RSSI (in the supply area of a small single brain artery) at University Hospital Graz between 2008 and 2013. We investigated associations between clinical and SVD features on MRI (STRIVE criteria) and recurrent cerebrovascular events, using multivariable Cox regression adjusted for age, sex, vascular risk factors and MRI parameters.

**Results:**

We analysed 332 consecutive patients (mean age 68 years, 36% women; median follow-up time 12 years). A recurrent ischaemic cerebrovascular event occurred in 70 patients (21.1%; 54 ischaemic strokes, 22 transient ischaemic attacks) and was mainly attributed to SVD (68%). 26 patients (7.8%) developed intracranial haemorrhage. In multivariable analysis, diabetes (HR 2.43, 95% CI 1.44–3.88), severe white matter hyperintensities (HR 1.97, 95% CI 1.14–3.41), and cerebral microbleeds (HR 1.89, 95% CI 1.32–3.14) on baseline MRI were related to recurrent ischaemic stroke/TIA, while presence of cerebral microbleeds increased the risk for intracranial haemorrhage (HR 3.25, 95% CI 1.39–7.59). A widely used SVD summary score indicated high risks of recurrent ischaemic (HR 1.22, 95% CI 1.01–1.49) and haemorrhagic cerebrovascular events (HR 1.57, 95% CI 1.11–2.22).

**Conclusion:**

Patients with RSSI have a substantial risk for recurrent cerebrovascular events—particularly those with coexisting chronic SVD features. Recurrent events are mainly related to SVD again.

## Introduction

Cerebral small vessel disease (SVD) is an important cause of ischaemic stroke as well as intracerebral haemorrhage, and is also associated with the development of vascular dementia, gait disturbance and depression [1, 2].

The clinical acute manifestation of SVD—lacunar stroke—accounts for approximately 20–25% of overall ischaemic stroke burden [3]. Recent small subcortical infarcts (RSSI) on diffusion-weighted brain MRI are the neuroimaging hallmark feature of acute lacunar stroke. However, it is well known that other conditions such as large artery disease or cardiogenic embolism (i.e., atrial fibrillation) can also (rarely) manifest as the neuroimaging appearance of an RSSI, and SVD and other potential stroke aetiologies can coexist [4, 5].

Interestingly, long-term outcome data of RSSI patients and potential risk factors for recurrent stroke and intracranial haemorrhage are limited.

Previous studies suggested an association of chronic SVD imaging markers and recurrent ischaemic stroke after lacunar stroke [6, 7]. Also, there are several studies focusing on the comparison of stroke recurrence and functional outcome between lacunar and non-lacunar stroke patients. These analyses show that the rate of recurrent ischaemic cerebrovascular events and mortality does not differ between different stroke subtypes in long-term observations [8–12]. However, these studies mostly defined the stroke subtype according to clinical rather than radiological characteristics or explicitly excluded patients with an RSSI pattern and coexisting atrial fibrillation or large artery disease (ipsilateral stenosis ≥ 50%). As not all RSSIs are caused by SVD alone and not all patients with a clinical lacunar syndrome show a corresponding RSSI on MRI, a certain mismatch between clinical and MRI-based definition of lacunar stroke exists in up to 20–25% of cases [13]. Moreover, little is known about the aetiologies of recurrent cerebrovascular events after an RSSI as there is just one study addressing this clinically highly relevant issue [6].

Therefore, the aim of this study was to identify clinical and imaging parameters that were associated with an increased risk of recurrent ischaemic stroke/transient ischaemic attack (TIA) and intracranial haemorrhage (ICH) in patients with an MRI-defined RSSI over a long-term follow-up period of 12 years [14].

Additional information on this regard as well as on the respective aetiologies of recurrent cerebrovascular events might be helpful in optimised follow-up care, secondary prevention and risk factor control in this often neglected subgroup of stroke patients.

## Methods

### Patient selection and data collection

**Fig. 1:**
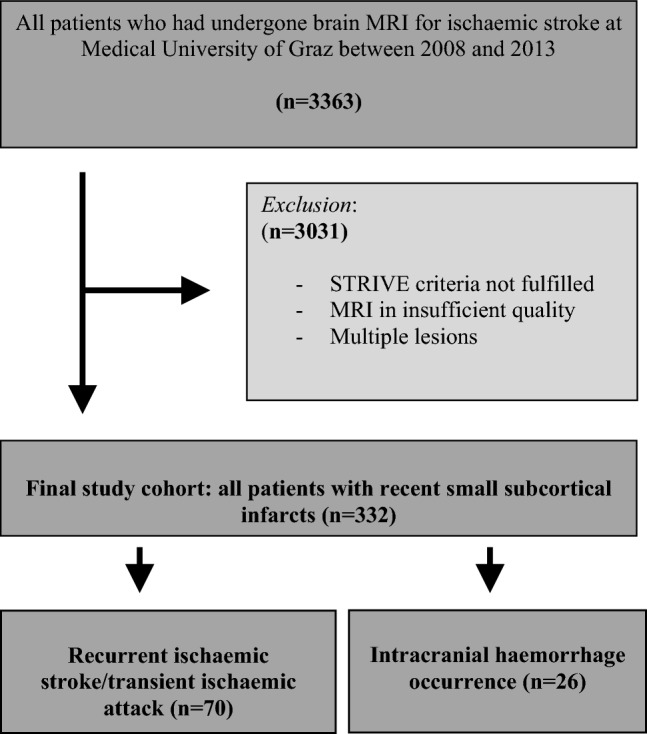
Patient selection and rate of recurrent cerebrovascular events

In this retrospective study, we considered all patients older than 18 years who were treated due to an ischaemic stroke between 2008 and 2013 at University Hospital Graz and had available brain MRI. The final study cohort comprised stroke patients with an MRI-confirmed single RSSI in the supply area of a small single perforating brain artery. RSSI was defined according to the standards for reporting vascular changes on neuroimaging (STRIVE) criteria [14]. Further exclusion criteria were the presence of more than one acute ischaemic lesion and insufficient quality of the available MRI scans (Fig. 1). We prespecified to not exclude patients with coexisting atrial fibrillation or upstream large artery disease (≥ 50% stenosis) [15].

Patient data including demographics, vascular risk factors (investigated vascular risk factors are listed in Tables 1 and 2), comorbidities, stroke severity (defined according to the National Institutes of Health Stroke Scale) [16] and recurrent (cerebro)vascular events were obtained from the electronic medical documentation system of our university hospital which also captures clinical data (including follow-up data) of all 11 public hospitals in the wider catchment area of the province of Styria, covering > 95% of all acute cerebro- and cardiovascular diseases treated in a hospital in this region [17]. Also, laboratory parameters (lipid profile, renal function parameters, HbA1c) at baseline and follow-up were assessed, if available.

**Table 1:** Demographic, clinical and radiological features of patients with and without recurrent ischaemic cerebrovascular events following RSSI

	All *N* = 332	Stroke/TIA recurrence *N* = 70	No Stroke/TIA recurrence *N* = 262	*P* Value	Odds ratio 95% CI
*Demographics*					
Age, years, mean, SD	67.7 ± 12.0	67.7 ± 10.1	67.7 ± 12.4	0.983	–
Male sex, *n* (%)	213 (64.2)	48 (68.6)	165 (63.0)	0.386	1.28 (0.73–2.25)
NIHSS admission, median (IQR)	3 (2)	3 (2)	3 (2)	0.337	–
NIHSS discharge, median (IQR)	1 (1)	2 (1)	1 (1)	0.212	–
Mortality during follow-up	64 (19.3)	12 (17.1)	52 (19.8)	0.610	0.84 (0.42–1.67)
*Vascular risk factors, n (%)*					
Arterial hypertension	279 (84.0)	62 (88.6)	217 (82.8)	0.244	1.61 (0.72–3.59)
Diabetes mellitus	89 (26.8)	26 (37.1)	63 (24.0)	0.028	1.86 (1.07–3.27)
Hyperlipidaemia	217 (65.4)	52 (74.3)	165 (63.0)	0.077	1.70 (0.94–3.07)
Smoking	107 (32.2)	24 (34.3)	83 (31.7)	0.679	1.13 (0.64–1.97)
Coronary disease	35 (10.5)	7 (10.0)	28 (10.7)	0.868	0.93 (0.38–2.22)
Atrial fibrillation	41 (12.3)	12 (17.1)	29 (11.1)	0.170	1.66 (0.80–3.46)
Ipsilateral large artery stenosis ≥ 50%	30 (9.0)	8 (11.4)	22 (8.4)	0.432	1.41 (0.59–3.31)
Peripheral artery disease	21 (6.3)	4 (5.7)	17 (6.5)	0.813	0.87 (0.28–2.68)
Previous stroke	71 (21.4)	19 (27.1)	52 (19.8)	0.186	1.50 (0.82–2.76)
Active cancer	9 (2.7)	2 (2.9)	7 (2.7	0.932	1.07 (0.22–5.28)
Chronic kidney disease GFR < 60 mL/min/L	76 (26.1)	19 (30.6)	57 (24.9)	0.360	1.33 (0.72–2.47)
Chronic kidney disease GFR < 45 mL/min/L	27 (8.1)	9 (12.9)	18 (6.9)	0.104	2.00 (0.86–4.67)
*Medication after index stroke, n (%)*					
Antithrombotics	295 (88.9)	59 (84.3)	236 (90.1)	0.171	0.59 (0.28–1.26)
Oral anticoagulation	37 (11.1)	11 (15.7)	26 (9.9)	0.171	1.69 (0.79–3.62)
Antihypertensives	258 (77.7)	60 (85.7)	198 (75.6)	0.070	1.93 (0.94–4.01)
Statins	195 (58.7)	49 (70.0)	146 (55.7)	0.031	1.83 (1.05–3.27)
Antidiabetics	77 (23.2)	22 (31.4)	55 (21.0)	0.066	1.73 (0.96–3.10)
*RSSI location, n (%)*					
Anterior Circulation	169 (50.9)	38 (54.3)	131 (50.0)	0.524	1.19 (0.70–2.02)
Basal ganglia	60 (18.1)	9 (12.9)	51 (19.5)	0.202	0.61 (0.28–1.31)
Internal capsule	45 (13.6)	15 (21.4)	30 (11.5)	0.030	2.11 (1.06–4.19)
Centrum semiovale	64 (19.3)	14 (20.0)	50 (19.1)	0.863	1.06 (0.55–2.06)
Posterior Circulation	163 (49.1)	32 (45.7)	131 (50.0)	0.524	0.84 (0.50–1.43)
Thalamus	76 (22.9)	13 (18.6)	63 (24.0)	0.333	0.72 (0.37–1.40)
Brainstem	87 (26.2)	19 (27.1)	68 (26.0)	0.841	1.06 (0.59–1.93)
*Cerebral small vessel disease-related imaging features*					
White matter hyperintensities*, median (IQR)	2 (2)	2 (2)	2 (2)	0.016	–
Severe white matter hyperintensities†, *n* (%)	188 (56.6)	50 (71.4)	138 (52.7)	0.005	2.29 (1.3–4.0)
Enlarged perivascular spaces**‡**, *n* (%)	214 (64.5)	44 (62.9)	170 (64.9)	0.865	0.93 (0.54–1.64)
Presence of one or more microbleed(s), *n* (%)	108 (32.5)	32 (45.7)	76 (29.0)	0.008	2.25 (1.26–3.98)
Presence of one or more lacune(s), *n* (%)	141 (42.5)	36 (51.4)	105 (40.1)	0.088	1.58 (0.93–2.68)
SVD summary score, median (IQR)	2 (2)	2 (2)	2 (2)	0.010	–
*Laboratory parameters (mean, standard deviation)*					
Low density lipoprotein, mg/dL	123.40 ± 36.12	123.2 ± 41.71	123.41 ± 34.84	0.971	–
Cholesterol, mg/dL	199.3 ± 50.79	199.4 ± 57.07	199.3 ± 48.92	0.988	–
GFR, mL/min/L	73.10 ± 21.96	73.92 ± 23.41	72.88 ± 21.60	0.743	–
Creatinine, mg/dL	1.07 ± 0.71	1.07 ± 0.51	1.08 ± 0.76	0.926	–
HbA1c, %	6.5 ± 2.7	7.5 ± 4.8	6.3 ± 1.6	0.014	–

*GFR* glomerular filtration rate; *SVD* cerebral small vessel disease.

*According to Fazekas Score.

†Severe white matter hyperintensities: deep Fazekas Score ≥ 2 or periventricular Fazekas Score 3.

‡Enlarged perivascular spaces: > 10 in one hemisphere in the basal ganglia.

**Table 2:** Demographic, clinical and radiological features of patients with and without intracranial haemorrhage following RSSI

	All *N* = 332	Intracranial Haemorrhage *N* = 26	No intracranial haemorrhage *N* = 306	*P* Value	Odds ratio 95% CI
*Demographics*					
Age, years, mean, SD	67.7 ± 12.0	69.4 ± 11.6	67.5 ± 12.0	0.445	–
Male sex, *n* (%)	213 (64.2)	17 (65.4)	196 (64.1)	0.892	1.06 (0.46–2.46)
NIHSS admission, median (IQR)	3 (2)	3 (2)	3 (2)	0.545	–
NIHSS discharge, median (IQR)	2 (1)	2 (1)	2 (1)	0.634	–
Mortality during follow-up	64 (19.3)	9 (34.9)	55 (18.0)	0.039	2.41 (1.02–5.70)
*Vascular risk factors, n (%)*					
Arterial hypertension	279 (84.0)	23 (88.5)	256 (83.7)	0.521	1.50 (0.43–5.18)
Hyperlipidaemia	217 (65.4)	18 (69.2)	199 (65.0)	0.666	1.21 (0.51–2.87)
Diabetes mellitus	89 (26.8)	5 (19.2)	84 (27.5)	0.364	0.62 (0.23–1.72)
Smoking	107 (32.2)	5 (19.2)	102 (33.3)	0.140	0.47 (0.18–1.30)
Coronary disease	35 (10.5)	2 (7.7)	33 (10.8)	0.672	0.69 (0.16–3.05)
Atrial fibrillation	41 (12.3)	4 (15.4)	37 (12.1)	0.624	1.32 (0.43–4.05)
Ipsilateral large artery stenosis ≥ 50%	30 (9.0)	1 (3.8)	29 (9.5)	0.336	0.38 (0.05–2.94)
Peripheral artery disease	21 (6.3)	0 (0.0)	21 (6.9)	0.168	–
Previous stroke	71 (21.4)	9 (34.6)	62 (20.3)	0.087	2.08 (0.89–4.90)
Active cancer	9 (2.7)	0 (0.0)	9 (2.9)	0.375	–
Chronic kidney disease GFR < 60 mL/min/L	76 (26.1)	5 (21.7)	71 (26.5)	0.618	0.77 (0.28–2.15)
Chronic kidney disease GFR < 45 mL/min/L	27 (8.1)	2 (7.7)	25 (8.2)	0.932	0.93 (0.21–4.19)
*Medication after index stroke, n (%)*					
Antithrombotics	295 (88.9)	24 (92.3)	271 (88.6)	0.560	1.55 (0.35–6.84)
Oral anticoagulation	37 (11.1)	2 (7.7)	35 (11.4)	0.560	0.65 (0.15–2.85)
Antihypertensives	258 (77.7)	22 (84.6)	236 (77.1)	0.378	1.63 (0.54–4.89)
Statins	195 (58.7)	17 (65.4)	178 (58.2)	0.473	1.36 (0.59–3.14)
Antidiabetics	77 (23.2)	4 (15.4)	73 (23.9)	0.326	0.58 (0.19–1.74)
*RSSI location, n (%)*					
Anterior Circulation	169 (50.9)	16 (61.5)	153 (50.0)	0.259	1.60 (0.70–3.64)
Basal ganglia	60 (18.1)	8 (30.8)	52 (17.0)	0.080	2.17 (0.90–5.23)
Internal capsule	45 (13.6)	2 (7.7)	43 (14.1)	0.363	0.51 (0.12–2.24)
Centrum semiovale	64 (19.3)	6 (23.1)	58 (19.0)	0.609	1.28 (0.49–3.34)
Posterior Circulation	163 (49.1)	10 (38.5)	153 (50.0)	0.259	0.63 (0.28–1.42)
Thalamus	76 (22.9)	4 (15.4)	72 (23.5)	0.343	0.59 (0.20–1.77)
Brainstem	87 (26.2)	6 (23.1)	81 (26.5)	0.706	0.83 (0.32–2.15)
*Cerebral small vessel disease-related imaging features*					
White matter hyperintensities*, median (IQR)	2 (2)	2 (1)	2 (2)	0.268	–
Severe white matter hyperintensities†, *n* (%)	188 (56.6)	20 (76.9)	168 (54.9)	0.030	2.74 (1.07–7.01)
Enlarged perivascular spaces**‡**, *n* (%)	214 (64.5)	19 (73.1)	195 (63.7)	0.442	1.70 (0.66–4.40)
Presence of one or more microbleed(s), *n* (%)	108 (32.5)	16 (61.5)	92 (30.1)	0.004	3.87 (1.64–9.07)
Presence of one or more lacune(s), *n* (%)	141 (42.5)	14 (53.8)	127 (41.5)	0.222	1.64 (0.74–3.67)
SVD summary score, median (IQR)	2 (2)	3 (2)	2 (2)	0.005	–
*Laboratory parameters (mean, standard deviation)*					
Low density lipoprotein, mg/dL	123.4 ± 36.12	104.13 ± 42.83	102.17 ± 38.21	0.846	–
Cholesterol, mg/dL	199.3 ± 50.79	203.50 ± 48.54	198.98 ± 50.93	0.664	–
GFR, mL/min/L	73.10 ± 21.96	71.95 ± 20.34	73.20 ± 22.13	0.793	–
Creatinine, mg/dL	1.07 ± 0.71	1.08 ± 0.69	1.08 ± 0.71	0.982	–
HbA1c	6.5 ± 2.7	5.8 ± 0.9	6.6 ± 2.8	0.262	–

*GFR* glomerular filtration rate; *SVD* cerebral small vessel disease.

*According to Fazekas Score.

†Severe white matter hyperintensities: deep Fazekas Score ≥ 2 or periventricular Fazekas Score 3.

‡Enlarged perivascular spaces: > 10 in one hemisphere in the basal ganglia.

All included RSSI patients received brain MRI, neurovascular ultrasound of extra- and intracranial vessels, an assessment of vascular risk factors as well as echocardiography and cardiac rhythm monitoring if indicated. Patients were specifically screened for the occurrence of recurrent ischaemic cerebrovascular events (transient ischaemic attack or stroke), intracranial haemorrhage or other vascular events (myocardial infarction, peripheral limb ischaemia) and overall mortality. Follow-up information was extracted until January 2023. Recurrent vascular events were defined as clinical stroke events (i.e., silent diffusion weighted imaging lesions were not considered). This information as well as the underlying aetiology was obtained from medical records (discharge letters, other medical documents, radiology reports, etc.) of the electronic medical documentation system of the province of Styria. Recurrent stroke/TIA aetiology was assessed according to the ASCOD classification [18].

## Imaging parameters

All included patients had received a 1.5 Tesla brain magnetic resonance imaging (MRI) at the time of the index event. MRI comprised a clinical standard protocol used in the workup of stroke patients (axial T2-weighted sequence, axial T2-weighted fluid-attenuated inversion-recovery sequence, gradient-echo T2*-weighted sequence and an axial diffusion-weighted sequence as well as intracranial time-of-flight magnetic resonance angiography) [19].

SVD features (presence and location of RSSI, presence and severity of white matter hyperintensities, presence of lacunes, presence of cerebral microbleeds, presence and number of enlarged perivascular spaces) were rated via cerebral MRI by two experienced neuroradiological experts blinded to outcome data and defined according to recent guidelines (STRIVE) [14]. White matter hyperintensities (WMH) were graded according to the Fazekas Score [20]. Severe WMH were defined as periventricular WMH 3 or deep WMH 2–3. Presence and numbers of enlarged perivascular spaces (EPVS) were evaluated in the basal ganglia. A number of more than 10 EPVS in the basal ganglia in one hemisphere was considered clinically significant [14, 21]. Furthermore, the presence and number of lacunes and cerebral microbleeds (CMB) were assessed. For CMB assessment, gradient echo T2*-weighted sequences were used.

We also applied a widely used SVD summary score combining the chronic SVD MRI features severity of white matter hyperintensities (WMH), presence of cerebral microbleeds (CMB), presence of lacunes of presumed vascular origin and enlarged perivascular spaces as defined above [21].

## Statistics

For statistical analyses, the software IBM SPSS (Version 29) was used. Continuous variables are presented as mean and standard deviation or as median and interquartile range; nominal variables are shown in absolute numbers and percentages. Pearson’s Chi-square test was used to compare nominal data. For the comparison of continuous data, Student’s *t* test or Mann–Whitney *U* test was used. Gaussian distribution was assessed with Kolmogorov–Smirnov test. To identify parameters that independently predicted the risk of recurrent ischaemic cerebrovascular events or occurrence of ICH, a multivariable Cox regression model was fitted. This analysis was adjusted for age, sex, vascular risk factors (arterial hypertension, hyperlipidaemia, diabetes mellitus) and MRI parameters of SVD. The multivariable model was calculated separately for individual SVD parameters (WMHs, cerebral microbleeds, lacunes) and the SVD summary score due to collinearity between the single SVD parameters and the SVD summary score. Besides age and sex, only parameters of interest that reached a *p* value < 0.1 in univariable analysis were included in the multivariable Cox regression model. Statistical significance was defined as a p value below 0.05.

## Results

The final study cohort included 332 patients (mean age 68 ± 12 years, 36% female). The median follow-up period was 12 years (IQR 3 years). The most common vascular risk factors were arterial hypertension (84.0%) and hyperlipidaemia (65.4%).

Baseline characteristics including vascular risk factors, demographics, baseline medication and imaging parameters are presented in Tables 1 and 2. RSSI were mainly located in the brainstem (*n* = 87, 26%), followed by thalamus (*n* = 76, 23%), centrum semiovale (*n* = 64, 19%), basal ganglia (*n* = 60, 18%) and the internal capsule (*n* = 45, 14%). At least one chronic SVD imaging feature was present in 286 patients (86.1%). 30 patients (9.0%) had a concomitant ipsilateral downstream arterial stenosis ≥ 50%, and 41 (12.3%) atrial fibrillation.

## Recurrent ischaemic cerebrovascular events

Recurrent ischaemic cerebrovascular events occurred in 70 patients (21.1%). 54 patients had ischaemic strokes; 22 patients had transient ischaemic attacks. The median time to ischaemic cerebrovascular event recurrence was 3 years (IQR = 6 years). Transient ischaemic attacks happened earlier (median 2 years, IQR = 3 years), than ischaemic stroke (median 4 years, IQR = 5 years). 16 patients (4.8%) suffered from multiple ischaemic cerebrovascular events during the follow-up period.

72% of recurrent strokes were due to SVD, whereas 13% were of cardiogenic origin and 9% occurred due to symptomatic large artery disease. 59% of transient ischaemic attacks were due to SVD, while 32% were attributed to cardiogenic origin and 9% to symptomatic large artery disease.

In univariable analysis, ischaemic cerebrovascular event recurrence was associated with diabetes mellitus (OR 1.86, 95% CI 1.07–3.27) and an increased HbA1c level (*p* = 0.014). Age, sex, other vascular risk factors as well as medication status and other laboratory parameters were not predictive in this context. Neither presence of concomitant atrial fibrillation nor large artery disease was associated with recurrent ischaemic cerebrovascular events (OR 1.66, 95% CI 0.80–3.46; OR 1.41, 95% CI 0.59–3.31 respectively, Table 1). Regarding brain MRI features, severe WMH and presence of CMB (Table 1) showed an association with recurrent ischaemic stroke/TIA (OR 2.29, 95% CI 1.30–4.0; OR 2.25, 95% CI 1.26–3.98, respectively). The median SVD summary score was higher in the subgroup of patients who had a recurrent ischaemic cerebrovascular event (*p* = 0.010).

In multivariable Cox regression diabetes, (HR 2.43, 95% CI 1.44–3.88), severe WMH (HR 1.97, 95% CI 1.14–3.41), CMB (HR 1.89, 95% CI 1.31–3.14) and the median SVD summary score (HR 1.22, 95% CI 1.01–1.49) were predictive for the occurrence of recurrent ischaemic cerebrovascular events (Table 3).

**Table 3:** Predictors for recurrent ischaemic cerebrovascular events and intracranial haemorrhage presented as multivariable cox regression model*

	Hazard Ratio	95% CI	*P* Value
*Recurrent ischaemic cerebrovascular events (n* = *70)*			
Age	1.006	0.984–1.028	0.585
Male sex	1.390	0.813–2.327	0.210
Arterial hypertension	1.415	0.643–3.110	0.388
Diabetes mellitus	**2.429**	**1.437**–**3.878**	** < 0.001**
Hyperlipidaemia	1.336	0.775–2.303	0.297
Severe white matter hyperintensities†	**1.970**	**1.138**–**3.410**	**0.015**
Presence of one or more cerebral microbleed(s)	**1.891**	**1.319**–**3.138**	**0.014**
Presence of one or more lacune(s)	1.287	0.783–2.114	0.320
SVD summary Score (median)	**1.224**	**1.005**–**1.492**	**0.044**
*Intracranial haemorrhage (n* = *26)*			
Age	1.039	0.992–1.088	0.106
Male sex	1.140	0.474–2.742	0.769
Arterial hypertension	1.117	0.315–3.964	0.864
Diabetes mellitus	0.942	0.342–2.597	0.909
Hyperlipidaemia	0.869	0.370–2.041	0.747
Severe white matter hyperintensities†	2.618	1.004–6.822	0.049
Presence of one or more cerebral microbleed(s)	**3.250**	**1.392**–**7.589**	**0.006**
Presence of one or more lacune(s)	1.593	0.703–3.605	0.264
SVD summary Score (median)	**1.571**	**1.111**–**2.222**	**0.011**

*SVD* cerebral small vessel disease.

*Adjusted for age, sex, arterial hypertension, diabetes mellitus, hyperlipidaemia and MRI parameters of SVD.

†Severe white matter hyperintensities: deep Fazekas Score ≥ 2 or periventricular Fazekas Score 3.

## Occurrence of intracranial haemorrhage

ICH occurred in 26 patients (7.8%) during the follow-up period and included intracerebral haemorrhage (*n* = 14; deep *n* = 13, lobar *n* = 1), subdural haemorrhage (*n* = 7) and subarachnoid haemorrhage (*n* = 5; aneurysmal *n* = 2, convexal *n* = 2, traumatic *n* = 1). The median time to the intracranial haemorrhage occurrence was 5 years (IQR = 5 years). Vascular risk factors, age, sex as well as medication status and laboratory parameters did not differ between patients with and without the occurrence of ICH in long-term follow up (Table 2).

Severe WMH (OR 2.74, 95% CI 1.07–7.01) and CMB (OR 3.87, 95% CI 1.64–9.07) were associated with ICH occurrence in univariable analysis. Moreover, the median SVD summary score was significantly higher in patients with ICH occurrence (3 vs. 2, *p* = 0.005). Maximal SVD summary score points of 4 were present in 31% of patients in this subgroup and were related to ICH (OR 3.04, 95% CI 1.24–7.47).

In the multivariable Cox regression model presence of CMB (HR 3.25, 95% CI 1.39–7.59, Table 3) and the median SVD summary score (HR 1.57, 95% CI 1.11–2.22) were predictive for ICH occurrence. Results did not change when only considering intracerebral haemorrhage subtype (*p* < 0.05 respectively).

## Other vascular events and mortality

Other arterial vascular events occurred in 21 patients (6.3%) and included myocardial infarction (*n* = 17, 5.1%) and peripheral limb ischaemia (*n* = 4, 1.2%).

The mortality rate during follow-up was 19.3% (*n* = 64). While the mortality rate was higher in the subgroup of patients suffering from an ICH during follow-up (HR 2.57, 95% CI 1.13–5.85), there was no association with ischaemic cerebrovascular event recurrence (HR 0.95, 95% CI 0.51–1.78) in univariable Cox regression analysis.

## Discussion

This retrospective study shows that RSSI patients have a substantial risk for recurrent cerebrovascular events over a long-term follow-up period (median 12 years). While diabetes mellitus was the only clinical risk factor that was related to recurrent ischaemic cerebrovascular events, the overall severity of chronic SVD features on baseline MRI (as assessed via a widely-used SVD summary score) [19] was associated with both recurrent stroke/TIA as well as ICH.

Regarding individual SVD MRI features, severity of WMH and CMB were associated with recurrent ischaemic cerebrovascular events, while presence of CMB was the sole factor related to ICH.

Contrary to previous studies [6, 7, 12] that mainly considered clinically-defined lacunar stroke patients (without consistently available brain MRI) or excluded patients with other coexisting aetiologies than SVD, we analysed a real-world stroke cohort with an MRI-confirmed RSSI according to the STRIVE criteria [14]. As we used an imaging selection approach (irrespective of the clinical stroke syndrome and results from further diagnostic workup), patients with coexisting atrial fibrillation or large artery disease were not excluded. Notably, presence of these two potential alternative stroke causes was not associated with recurrent cerebrovascular events.

Due to the smaller lesion size and often low initial stroke severity, lacunar stroke/RSSI is frequently considered as a more benign subtype of ischaemic stroke. In contradiction to this assumption, recent studies showed that even though the risk for recurrent vascular events after RSSI in shorter time observations is lower than in other stroke subtypes, there is no significant difference in long-term outcome [8, 10, 12]. The results of our analysis support these findings with a high rate of recurrent ischaemic cerebrovascular events of 21% and also a remarkable rate of future ICH of 8% following RSSI in a median follow-up period of 12 years. Not surprisingly, ICH occurrence was also related to an increased mortality rate.

Only few previous studies focusing on recurrent cerebrovascular events in RSSI patients can be found in the current literature. A substudy of the Secondary Prevention in Small Subcortical Strokes trial (SPS3) showed a higher rate of stroke recurrence in patients with concomitant diabetes and metabolic syndrome [22]. Our study also identified diabetes as a risk factor for recurrent ischaemic cerebrovascular events in multivariable analysis. We could not identify arterial hypertension as predictor for recurrent cerebrovascular events. Notably, the use of antihypertensive medication was higher in numbers in patients with recurrent ischaemic cerebrovascular events than in those without. Even though this association did not reach statistical significance, it could indicate a higher rate of ongoing or more severe hypertension in patients with recurrent stroke after RSSI.

Few studies investigated the impact of neuroimaging SVD parameters on the frequency of recurrent cerebrovascular events in RSSI cohorts [6, 7]. Imaizumi et al. showed that severe WMH (Fazekas scale 2–3) and the presence of CMB correlated with recurrent lacunar stroke in a lacunar stroke cohort of 305 patients over a follow-up time of about 4 years [6]. Our investigation confirmed these results.

Our analysis also included a widely used SVD summary score, which was evidently higher in the subgroup of patients who developed recurrent cerebrovascular events. These results indicate that the overall SVD burden on MRI identifies patients at risk for stroke recurrence and that a more stringent follow-up regime in this patient cohort during long-time follow-up might be justified. Similar results were already shown by a previous work in a short-term follow-up study of three months [7]. It is of note that due to the short follow-up period the number of recurrent events was significantly lower than in our analysis (2.9% recurrent ischaemic stroke in a population of 599 patients). Notably, in this work, acute lacunar infarcts were also defined according to STRIVE criteria but contrary to our approach patients whose index stroke was potentially caused by cardiogenic embolism or other non-SVD-related causes were excluded. Furthermore, our analysis also focused on the aetiologies of recurrent ischaemic cerebrovascular events and showed that 72% of recurrent stroke and 59% of TIA were caused by SVD again. The systematic assessment of the aetiologies of recurrent cerebrovascular events after RSSI in current literature is limited [6] with one single study showing that lacunar stroke patients have a higher chance of lacunar stroke reoccurrence compared to other stroke subtypes [23].

Studies focusing on the occurrence of intracranial or intracerebral haemorrhage after RSSI or lacunar infarction are limited to date. The only available study showed that severe WMH also increase the risk of deep intracerebral haemorrhage after lacunar infarction [6]. Albeit ICH numbers might have been too small in our cohort, we did not find such an association in our study. After correction for age and vascular risk factors, CMB remained the only SVD-related imaging feature related to ICH occurrence. However, the overall SVD burden (based on a summary score) was also strongly associated with ICH occurrence. One could argue that the presence of CMB would be a major factor in this equation as CMB was the only imaging parameter that was associated with ICH occurrence in multivariable analysis. Also, a correlation of the presence of CMB and ICH occurrence has been shown in studies before [24]. However, the number of patients with a SVD summary score of 4 was particularly high in this subgroup of patients (31% vs. 20% in the recurrent ischaemic event subgroup and 12% in the overall study cohort). This assumption is supported by Goeldlin et al. who showed a higher SVD summary score in intracerebral haemorrhage patients than in lacunar stroke patients [7]. Even though we did not exclude other forms of intracranial haemorrhage such as subdural or subarachnoid bleeding, these associations remained significant.

Interestingly, we could identify both the presence of CMB and an increased SVD summary score as predictors for recurrent ischaemic and haemorrhagic events. This could indicate shared risk factors and underlying mechanisms in pathophysiology and might have implications on secondary prevention strategies in clinical practice.

The strengths of this study are the long follow-up period and the availability of MRI scans that made it possible to correct for SVD imaging parameters, which was often limited in previous studies on this topic. Moreover, the RSSI cohort of this study was defined based on MRI characteristics according to the STRIVE criteria and patients with coexisting atrial fibrillation or large artery disease were not a-priori excluded – reflecting a real-world stroke cohort with often coexisting risk factors and aetiologies. This work also benefits from a thorough analysis on aetiologies of recurrent cerebrovascular events and demonstrates that most ischaemic re-events after an RSSI are caused by SVD again.

This study also has some limitations which are mostly due to its retrospective study design as parameters of interest were not always consistently documented in follow-up. In this context the lack of systematic follow-up visits, which could assess risk factor management, medication adherence and blood pressure control including potential episodes of uncontrolled blood pressure is a clear limitation. Also, we cannot rule out possible bias in the reporting of smoking status due to missing documentation or underreporting of the patients during follow-up.

However, as the medical electronic database of our hospital covers all acute neurological units in our catchment area, it is very unlikely that recurrent cerebrovascular events have been missed in our analysis, which was the main focus of this study. Also, the lack of systematic follow-up imaging studies did not allow us to investigate SVD progression over time and its impact on cerebrovascular events.

## Data Availability

The data acquired for this study are available from the corresponding author upon reasonable request.

## Abbreviations

CMB: Cerebral microbleeds
EPVS: Enlarged perivascular spaces
ICH: Intracranial haemorrhage
SVD: Cerebral small vessel disease
WMH: White matter hyperintensities
